# Alternative splicing of basic chitinase gene *PR3b* in the low-nicotine mutants of *Nicotiana tabacum* L. cv. Burley 21

**DOI:** 10.1093/jxb/erw345

**Published:** 2016-09-23

**Authors:** Haoran Ma, Feng Wang, Wenjing Wang, Guoying Yin, Dingyu Zhang, Yongqiang Ding, Michael P. Timko, Hongbo Zhang

**Affiliations:** ^1^Tobacco Research Institute, Chinese Academy of Agricultural Sciences, Qingdao 266101, China; ^2^College of Agronomy and Biotechnology, Southwest University, Chongqing 400716, China; ^3^Department of Biology, University of Virginia, Charlottesville, Virginia 22904, USA

**Keywords:** Alternative splicing, ethylene, jasmonate, low-nicotine mutant, *PR3b*, tobacco.

## Abstract

Two unlinked semi-dominant loci, *A* (*NIC1*) and *B* (*NIC2*), control nicotine and related alkaloid biosynthesis in Burley tobaccos. Mutations in either or both loci (*nic1* and *nic2*) lead to low nicotine phenotypes with altered environmental stress responses. Here we show that the transcripts derived from the pathogenesis-related (PR) protein gene *PR3b* are alternatively spliced to a greater extent in the *nic1* and *nic2* mutants of Burley 21 tobacco and the *nic1nic2* double mutant. The alternative splicing results in a deletion of 65 nucleotides and introduces a premature stop codon into the coding region of *PR3b* that leads to a significant reduction of PR3b specific chitinase activity. Assays of *PR3b* splicing in F_2_ individuals derived from crosses between *nic1* and *nic2* mutants and wild-type plants showed that the splicing phenotype is controlled by the *NIC1* and *NIC2* loci, even though *NIC1* and *NIC2* are unlinked loci. Moreover, the transcriptional analyses showed that the splicing patterns of *PR3b* in the low-nicotine mutants were differentially regulated by jasmonate (JA) and ethylene (ET). These data suggest that the *NIC1* and *NIC2* loci display differential roles in regulating the alternative splicing of *PR3b* in Burley 21. The findings in this study have provided valuable information for extending our understanding of the broader effects of the low-nicotine mutants of Burley 21 and the mechanism by which JA and ET signalling pathways post-transcriptionally regulate the activity of PR3b protein.

## Highlight

Alternative splicing of the basic chitinase gene *PR3b* requires genes in the *NIC* loci of low-nicotine mutants of Burley 21 tobacco.

## Introduction

Nicotine is a natural compound used for defence against attack by insect herbivores in members of the genus *Nicotiana* and is the predominant alkaloid in most cultivated commercial tobacco (*Nicotiana tabacum* L.) varieties (Baldwin, 2001; Steppuhn *et al.*, 2008; Kumar *et al.*, 2014; Sears *et al.*, 2014; Shitan *et al.*, 2015). The formation of nicotine begins with ornithine and/or arginine and involves the key catalytic enzymes, PMT (putrescine *N*-methyltransferase), ODC (ornithine decarboxylase), QPT (quinolinate phosphoribosyltransferase), MPO (*N*-methylputrescine oxidase), A622 (isoflavone reductase-like), BBL (berberine bridge enzyme-like), and MATE (multidrug and toxic compound extrusion) (Hibi *et al.*, 1994; Sinclair *et al.*, 2000; Heim *et al.*, 2007; Deboer *et al.*, 2009; Kajikawa *et al.*, 2011; Dewey and Xie, 2013; Lewis *et al.*, 2015). The process of nicotine formation takes place in the roots and is regulated by the developmental stage of the plant, phytohormonal signals, and environmental factors (Baldwin, 1998; Xu and Timko, 2004; Shi *et al.*, 2006; Li *et al.*, 2007).

The phytohormone jasmonate (JA) is a major regulator of nicotine synthesis, and an increase in endogenous JA level or an exogenous application of JA or MeJA (methyl jasmonate) rapidly increases the transcript levels of genes encoding enzymes of nicotine biosynthesis (e.g. PMT, QPT), to promote nicotine synthesis (Baldwin, 1998; Imanishi *et al.*, 1998; Shoji *et al.*, 2000*b*; Goossens *et al.*, 2003; Saedler and Baldwin, 2004; Xu and Timko, 2004; Cane *et al.*, 2005; Shoji *et al.*, 2008; Zhang *et al.*, 2012). A number of key JA-signalling components have been demonstrated to be involved in the fine-tuning of JA-induced nicotine synthetic genes (Shoji *et al.*, 2008; Shoji and Hashimoto, 2011; Zhang *et al.*, 2012; Woldemariam *et al.*, 2013). Interestingly, several studies also suggested that the gaseous phytohormone ethylene (ET) plays a negative role in nicotine synthesis by suppressing the expression of nicotine synthetic genes (Shoji *et al.*, 2000*a*; Winz and Baldwin, 2001; Xu and Timko, 2004). Moreover, members of the plant-specific transcription factor family known as ERFs (ethylene response factors) have been shown to play critical roles in JA/ET signalling and in the regulation of nicotine synthesis (Guo and Ecker, 2004; Gutterson and Reuber, 2004; Shoji *et al.*, 2010; De Boer *et al.*, 2011; Sears *et al.*, 2014).

ERF proteins were first identified as transcription factors regulating plant pathogen resistance in tobacco (Ohme-Takagi and Shinshi, 1995). Thereafter, a large number of ERF transcription factors have been identified as regulators functioning in plant stress tolerance, pathogen resistance, secondary metabolism, and signal transduction of phytohormones [e.g. JA, ET, salicylic acid (SA)] (Chakravarthy *et al.*, 2003; Lorenzo *et al.*, 2003; Gutterson and Reuber, 2004). A major target of ERF proteins is the GCC-box in the promoter regions of pathogenesis-related (PR) protein genes that function in plant pathogen resistance (Ohme-Takagi and Shinshi, 1995; Chakravarthy *et al.*, 2003; Zhang *et al.*, 2004). A number of ERFs were shown to be co-ordinated by JA and ET signalling pathways to regulate pathogen resistance and secondary metabolism (Lorenzo *et al.*, 2003; Gutterson and Reuber, 2004; Zhang *et al.*, 2004; Pre *et al.*, 2008; Shoji *et al.*, 2010). On the other hand, ERF transcription factors could synergistically or antagonistically co-operate with other regulators to co-ordinate the JA and ET signalling (Lorenzo *et al.*, 2003; Pre *et al.*, 2008; Zarei *et al.*, 2011). Therefore, the integrative framework involving ERF transcription factors and their target genes is of great importance for dissecting JA- and/or ET-mediated regulation in plants.

The low-alkaloid mutant of Burley 21 (LA Burley 21) is a genetically stable breeding line that was developed in the early 1930s from Cuban tobacco cigar varieties having very low nicotine content (Valleau, 1949; Legg *et al.*, 1970). Genetic studies in LA Burley 21 demonstrated that nicotine leve1s are controlled by two unlinked semi-dominant loci, *A* and *B* (also known as *NIC1* and *NIC2*) (Legg *et al.*, 1969; Legg and Collins, 1971; Hibi *et al.*, 1994). These loci act synergistically in regulating nicotine synthesis (Legg *et al.*, 1969; Hibi *et al.*, 1994; Kidd *et al.*, 2006). The transcript level of *PMT* was established as a marker widely used in nicotine synthesis studies and subsequent transcriptional analyses identified a set of nicotine synthesis genes that were down-regulated in mutant alleles of *NIC1* and *NIC2* (Hibi *et al.*, 1994; Reed and Jelesko, 2004; Cane *et al.*, 2005; Shoji *et al.*, 2010). Recent studies have shown that the *NIC2* locus contains a cluster of ERF transcription factors (Shoji *et al.*, 2010; Shoji and Hashimoto, 2014). Furthermore, research using a fluorescent differential display (FDD) screen provided evidence that the *NIC* loci regulated a large number of stress-responsive genes but only a small portion of these genes were involved in nicotine synthesis (Kidd *et al.*, 2006). This may indicate a broad regulatory function of *NIC* loci.

While the *NIC2* locus is comprised of a cluster of ERF-transcription-factor-coding genes, the transcriptional regulation of PR protein genes in the low-nicotine mutants of Burley 21 is less well understood (Lorenzo *et al.*, 2003; Guo and Ecker, 2004; Gutterson and Reuber, 2004; Pre *et al.*, 2008; Shoji *et al.*, 2010; Shoji and Hashimoto, 2014). In this study, the expression patterns of a set of PR protein genes were analysed in the low-nicotine mutants *nic1*, *nic2*, and in the double mutant *nic1nic2*. We identified that *PR3b*, a basic chitinase gene, is alternatively spliced in plants containing mutant alleles of the *NIC1* and *NIC2* loci. This splicing resulted in a deletion of 65bp nucleotides and introduced a premature stop codon into the coding region of the *PR3b* mRNA which, in turn, changed the enzyme-specific activity of PR3b. The genetic linkage between *PR3b* splicing and the *NIC* loci and the regulation of *PR3b* splicing by phytohormone JA and ET were also further investigated. Findings in this study indicate a novel regulatory pattern of *PR3b* and provide new insights into the genetic basis of the low-nicotine mutants of Burley 21.

## Materials and methods

### Plant materials

Plant materials used in this study include wild-type *N. tabacum* cv. Burley 21 and low-nicotine Burley 21 mutants (*nic1*, *nic2*, and *nic1nic2*). For the transcriptional assay, seeds of the desired tobacco varieties were germinated and grown for 1 week on plates with 1/2 strength Murashige & Skoog (1/2 MS) complete medium for which a commercial product of the MS basal salt mixture (Duchefa, Netherland) was used. Their seedlings were then transferred into sterile hydroponic culture chambers supplied with 200ml liquid 1/2 MS complete medium for four plants each and cultured in a growth room at 25 °C under 2 500 lx light intensity supplied by cool-white fluorescent tubes (Phillips, USA) and a 14h light/10h dark photoperiod. The hydroponic culture medium was changed weekly with fresh medium. For inductive phytohormone treatment, 5-week-old plants were treated using liquid 1/2 MS complete medium containing 50 μM ACC (1-aminocyclopropane-1-carboxylic acid; Sigma-Aldrich), or 50 μM JA (MeJA; Sigma-Aldrich) or the combination of 50 μM ACC and 50 μM JA for 24h. The roots and leaves of tobacco seedlings were collected separately for total RNAs extraction. Roots and leaves from seedlings of control treatment with phytohormone-free medium were collected as controls. 

For genetic assay, the low-nicotine *nic1* and *nic2* mutant plants were fertilized with pollen from wild type Burley 21 to obtain the F_1_ plants, and the F_1_ plants were self-pollinated to generate F_2_ populations. Individual F_2_ seedlings were initially hydroponically cultured as described above to collect root samples for RNA preparation and then transplanted to the field for the preparation of leaf samples used for the measurement of nicotine content.

### Transcriptional analyses using reverse transcription PCR (RT-PCR)

Total RNAs were extracted using the TRIZOL reagent (Invitrogen) according to the manufacturer’s instructions. First-strand cDNAs were synthesized from 5 μg of DNase I-treated total RNAs using a Cloned AMV First-Strand cDNA Synthesis Kit (Invitrogen) with oligo(dT)_20_. Aliquots containing reverse-transcribed products from 100ng of total RNAs were used as templates for each semi-quantitative RT-PCR or quantitative RT-PCR (qRT-PCR) reaction. Primers used for RT-PCR and qRT-PCR were as follows: 5′-AAAATGGCACTTCTGAACAC-3′ and 5′-CCAGGCTTAATAGAGTTGGA-3′ for *PMT1*; 5′-ACGAC CAGGTAGCAGCCTAT-3′’ and 5′-TTAGCAGCCGTCATGAA ATC-3′ for *PR1a*; 5′-TGCAACAATGGGTGGTATTT-3′ and 5′-G GAATCAAAGGGATGTTGCT-3′ for *PR1b*; 5′-AAGCTGGTTT GGGAAACAAC-3′ and 5′-AAACCACCTAGCATCGTTCC-3′ for *PR2b*; 5′-AGGAGGTGGAATCAGTGGAC-3′ and 5′-TGACATTA GCACTTGCTTTGG-3′ for *PR3b*; 5′-GGGTAAACCACCAAAC ACCT-3′ and 5′-GGGAAAGTGATCGGAATGTT-3′ for *PR5*; 5′-CC ACACAGGTGTGATGGTTG-3′ and 5′-GTGGCTAACACCATCA CCAG-3′ for *Actin*. 5′-GAGCAATTCAGAAAATTTCAAGAGG-3′ (upstream of the splicing region) was combined, respectively, with 5′-CATTACCCGCGGCTGTCTTGGCTG-3′ (for native *PR3b* and specific to the fragment to be excised in the splicing) and 5′-CTTGCTTTGGTTTGTGTCCACTG-3′ (for spliced *PR3b* and specific to the spliced junction) in order to quantify the transcript levels of native and spliced *PR3b* specifically. Three independent biological replicates were performed for all experiments.

For the semi-quantitative RT-PCR assay, the PCR amplification program was 25 cycles of 1min at 94 °C, 40s at 58 °C, and 40s at 72 °C. The PCR products were separated using a 1% agarose gel, stained with ethidium bromide, and visualized under UV light. qRT-PCR reactions were performed on a Stratagene Mx3000P™ quantitative PCR system (Stratagene, USA) using GoTaq® qPCR Master Mix (Promega). *Actin* was used as the internal control. The relative transcripts were obtained by calibrating the threshold cycles of genes of interest with that of *Actin* using the equation 2^(–ΔΔCT)^, as previously described by Zhang *et al.* (2012), where C_T_ is the cycle number of the threshold point at which fluorescence is detectable.

### Rapid amplification of cDNA ends (RACE) of spliced PR3b

Nested PCR method was applied for the RACE PCR using a Smarter RACE kit (Clontech, USA) except that the PCR reagents of a Phire Plant Direct kit (Thermo, USA) were used for the amplification. cDNAs were synthesized with total RNAs from the roots of *nic* mutants according to the manufacturer’s instruction. 5′-RACE was initially amplified at an annealing temperature of 56 °C with the gene-specific primer 5′-TGACATTAGCACTTGCTTTGG-3′ (downstream of the splicing region) and the universal primer provided by the Smarter RACE kit, and then amplified at an annealing temperature of 60 °C with primer 5′-CTTGCTTTGGTTTGTGTCCACTG-3′ (specific to the spliced junction of *PR3b*) and the universal primer. 3′-RACE was done in a similar way. The gene-specific primer for the initial amplification is 5′-AGGAGGTGGAATCAGTGGAC-3′ (upstream of the splicing region), and that for the second round amplification is 5′-CAGTGGACACAAACCAAAGCAAG-3′ (specific to the spliced junction of *PR3b*). The PCR products were ligated into *Eco*RV-digested pBlueScript II SK+ vector and then sequenced.

### Comparison of the RT-PCR and genomic DNA PCR products of PR3b

Genomic DNA was extracted from tobacco roots using the CTAB method, as described by Zhang *et al.* (2012). 100ng genomic DNA was used as a template for PCR amplification of the *PR3b* fragment. The PCR amplification program was 30 cycles of 1min at 94 °C, 40s at 58 °C, and 40s at 72 °C.

RT-PCR and genomic DNA PCR amplification products of the *PR3b* fragments were compared by electrophoresis on a 1.5% (w/v) agarose gel. The amplifications products were visualized by staining with ethidium bromide and exposure under UV light. The 1kb DNA Ladder (Invitrogen, USA) was used as a DNA molecular weight marker.

### Gel extraction and abundance estimation of PR3b isoforms

For sequencing of the amplified *PR3b* DNA or cDNA fragments, the corresponding PCR products were first separated on a 1% agarose gel. The gel fragments containing the amplification products of both native and alternatively spliced *PR3b* fragments were purified using a Gel Extraction Kit (BBI), ligated into the pBlueScript II SK+ vector (Stratagene), and sequenced using M13 primers. Fifty positive clones of each sample were sequenced, and the abundance of *PR3b* isoforms estimated.

### Bioactivity assay of spliced PR3b variant

To obtain proteins for the enzymatic assay, the coding sequences of native *PR3b* and alternatively spliced *PR3b* were amplified with the same 5′-end primer 5′-AAA*GGATCC*ATGAGCATTAAGCTATCTT-3′ (restriction site is italicized) and specific 3′-end primers 5′-AACAGCACCCCTGATAGC-3′ (for native *PR3b*) and 5′-TTCCAAAGCATGACACCTC-3′ (for spliced *PR3b*), and cloned in-frame with the glutathione *S*-transferase (GST) tag coding region in pGEX-4T-2 vector (Novagen) through restriction sites *Bam*HI and *Sma*I. Then, the protein expression vectors were transformed into *E. coli* BL21 cells to induce prokaryotic protein expression by treating cells with 1mM IPTG for 3h at 37 °C. The recombinant proteins were purified using GST affinity column chromatography according to the manufacturer’s protocol (Invitrogen) and dialysed against the dialysing buffer (50mM sodium phosphate, 10% glycerol, pH 6.5). The empty pGEX-4T-2 vector was used to produce control GST protein in the same procedure. The enzyme-specific activity was determined with a fluorimetric Chitinase Assay Kit (Sigma CS1030) with 4-methylumbelliferyl β-d-*N*, *N* ′, *N* ′-triacetylchitotriose [4MU-GlcNAc_3_] as substrate (Brotman *et al.*, 2012) according to the manufacturer’s introduction. After incubation for 1h at 37 °C, fluorescence of the reaction mixture was measured by a fluorescence spectrophotometer (excitation at 360nm, emission at 450nm).

### Leaf nicotine measurement

Dry leaf samples were subjected to alkaloids extraction as previously described previously (Goossens *et al.*, 2003; Zhang *et al.*, 2012) with minor modification. Briefly, 10mg of homogenized dry leaf sample was soaked in 1ml of 10% NaOH (w/v) for 20min and then extracted by vortexing with an equivalent volume of dichloromethane. The organic layer was collected after centrifugation. The nicotine content was measured on an Agilent Technologies 7890A Chromatograph equipped with a DB 5 MS column and Agilent Technologies 5975C inert MSD detector with helium as the carrier gas. The column temperature was held at 100 °C for 5min, increased to 210 °C at an increment of 50 °C min^–1^, and then held at 210 °C for 4min. The ion source temperature was 230 °C and the quadrupole temperature was 150 °C. Nicotine from Sigma–Aldrich was used as the standard control.

### Gene accessions

The NCBI accession numbers for the *N. tabacum* genes mentioned in this article are as follows: *PMT1* (AF126810), *PR1a* (X12737), *PR1b* (X66942), *PR2b* (M59442), *PR3b* (Z11564), *PR5* (M29279), and *Actin* (X63603).

## Results

### JA/ET-induced expression patterns of a set of PR protein genes in the roots of Burley 21 tobacco

It is well documented that PR protein genes functioning in plant pathogen resistance are regulatory targets of ERF transcription factors (Chakravarthy *et al.*, 2003; Guo and Ecker, 2004; Gutterson and Reuber, 2004). The *NIC1* and *NIC2* loci integrate the regulation of nicotine biosynthetic genes and stress-responsive genes (Hibi *et al.*, 1994; Cane *et al.*, 2005; Kidd *et al.*, 2006), and the *NIC2* locus contains a cluster of ERF transcription factors (Shoji *et al.*, 2010). Therefore, we hypothesized that mutations in *NIC1* or *NIC2* are likely to alter the PR protein gene expression patterns. To explore this possibility, we first selected a set of PR protein genes known to be regulated by the JA and/or ET pathways and ERF transcription factors (Ohme-Takagi and Shinshi, 1995; Chakravarthy *et al.*, 2003; Lorenzo *et al.*, 2003; Zhang *et al.*, 2004; van Loon *et al.*, 2006), and tested their induction by JA and/or ACC (1-aminocyclopropane-1-carboxylic acid; the immediate precursor of ethylene) in the roots of wild-type Burley 21 tobacco. The PR protein genes selected included **PR1a* (acidic PR1 gene*), *PR1b* (basic PR1 gene), *PR2b* (basic beta-1,3-glucanase gene), *PR3b* (basic chitinase III gene), and *PR5* (osmotin gene). The expression level of *PMT1*, a well characterized gene involved in nicotine biosynthesis (Riechers and Timko, 1999), was also analysed as a control.

**Fig. 1.**
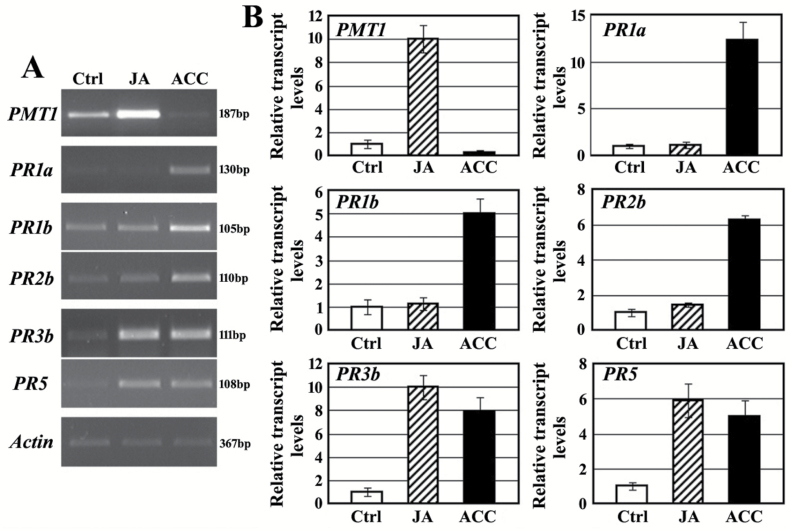
Induced expression patterns of the nicotine synthetic gene *PMT1* and PR protein genes in the roots of wild-type tobacco Burley 21. (A) Expression pattern assay by semi-quantitative RT-PCR. The representative results of three independent replicates are shown. The sizes of the amplified products are indicated on the right. (B) Transcript levels of *PMT1* and PR protein genes based on qRT-PCR analysis. Ctrl indicates untreated control. JA and ACC indicate treatment with JA and ACC, respectively. The transcription level of each gene in the Ctrl is set as ‘1’. *Actin* was used as an internal control for both semi-quantitative RT-PCR and qRT-PCR.

We analysed the expression patterns of the selected PR protein genes in the roots of wild-type tobacco treated for 24h with JA or ACC. As shown in Fig. 1A, the designed primers could specifically amplify the target fragments of PR protein genes in the roots of wild-type Burley 21. The expression of *PMT1* was dramatically induced by JA but inhibited by ACC; the expression of *PR1a*, *PR1b*, and *PR2b* could only be induced by ACC treatment and the expression of *PR3b* and *PR5* was induced by both JA and ACC treatments (Fig. 1A, B). These findings established a preliminary regulatory relationship between the nicotine biosynthetic pathway and the regulation of PR protein genes by the JA and ET signalling pathways in Burley 21 tobacco.

### PR3b is alternatively spliced in low-nicotine mutants of Burley 21

**Fig. 2.**
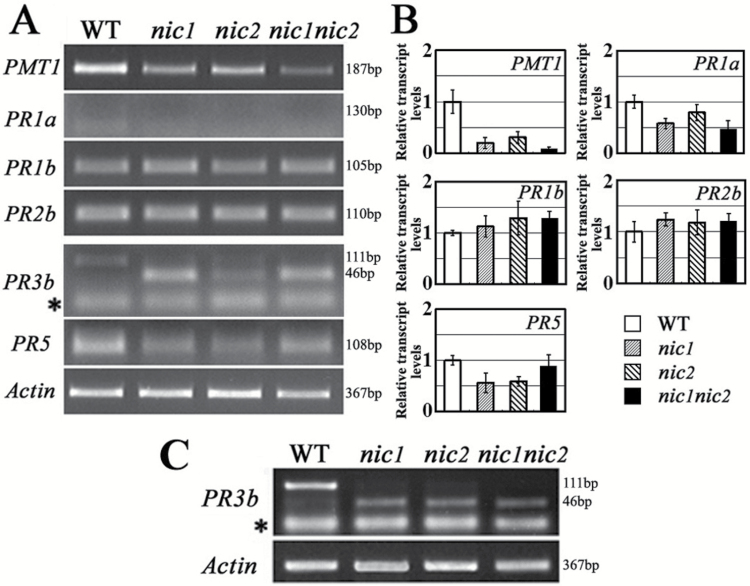
Transcription patterns of *PMT1* and PR protein genes in the wild type and in low-nicotine mutants of Burley 21. (A) Expression patterns of *PMT1* and PR protein genes in roots by semi-quantitative RT-PCR assay. (B) Expression levels of *PMT1* and PR protein genes determined by qRT-PCR. The transcription level of each gene in the Ctrl is set as ‘1’. (C) Transcripts of *PR3b* gene in leaves. (A, C) The representative results of three independent replicates and the sizes of the amplified products are indicated on the right. WT indicates wild type Burley 21. *nic1*, and *nic2* indicate low-nicotine mutant alleles of *NIC1* and *NIC2*, and *nic1nic2* indicates the double mutant. The asterisk indicates primer dimers in the amplification products of the *PR3b* gene. *Actin* was used as an internal control.

**Fig. 3.**
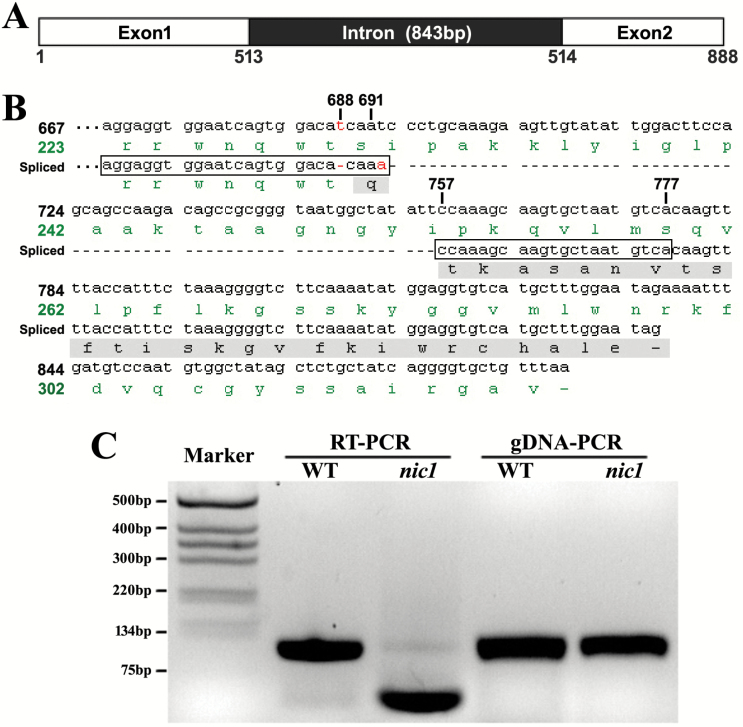
Characterization of the alternative splicing of *PR3b*. (A) Schematic gene structure of *PR3b*. The details are shown in Supplementary Fig. S1. (B) Alignment of the alternatively spliced *PR3b* fragment (Spliced) against the native *PR3b* cDNA (sequence with labelled nucleotide numbers). The numbers indicate the position of the indicated nucleotide at the native *PR3b* coding region. The alternative spliced region is highlighted by rectangles and dashes between the rectangles indicate the excised region by the alternative splicing. Positions corresponding to the single-nucleotide mutation sites are indicated by red characters. The amino acid sequences in green are deduced from the native *PR3b* cDNA. The amino acid sequence highlighted by the grey bar shows the coding change region caused by alternatively splicing of *PR3b*; the dash in the amino acid sequence indicates the position of the stop codon. (C) Comparison of RT-PCR and genomic DNA PCR (gDNA-PCR) products amplified using the same primers. Marker indicates the DNA molecular marker. WT, wild type; *nic1*, low-nicotine mutant *nic1*.

To investigate the potential roles of *NIC* loci in regulating PR protein genes, transcript levels of the selected PR protein genes were comparatively analysed in the untreated low-nicotine mutants (*nic1*, *nic2*, and *nic1nic2*) as well as the wild-type control. RT-PCR assays revealed that the transcript level of *PMT1* was down-regulated to different extents in the mutants (Fig. 2A, B). No obvious transcriptional differences were observed for *PR1b* and *PR2b* in the wild type or low-nicotine mutants of Burley 21, whereas the transcript levels of *PR1a* and *PR5* were slightly down-regulated in the low-nicotine mutants (Fig. 2A, B). While all the above amplifications gave the expected specific products, the amplification products of *PR3b* from the low-nicotine mutants showed two distinct bands in the gel: a faint band of the same size amplified from wild-type Burley 21 and a smaller, more intense band which is hard to visualize in the wild-type control (Fig. 2A). The amplification of *PR3b* transcripts from leaf tissue gave a similar result to the roots (Fig. 2C). To establish whether the smaller band was an alternative transcript of *PR3b* or a non-specifically amplified fragment, the *PR3b* RT-PCR amplification products were sequenced. This revealed that the larger product is the amplification of native *PR3b* transcript while the smaller one is an amplification of an alternatively spliced transcript of *PR3b* (Fig. 3B). These data implied a difference in the post-transcriptional regulation of *PR3b* transcripts between the wild type and the low-nicotine mutants of Burley 21.

### Characterization of the alternatively spliced transcripts of PR3b

In order to reveal further information about the alternatively spliced transcript of *PR3b*, we compared its nucleotide sequence with the native *PR3b* cDNA sequence using DNA sequence alignment analysis. The genomic sequence of *PR3b* contains two exons and one intron (Fig. 3A; see Supplementary Fig. S1 at *JXB* online), and the alternative splicing occurs in the region of exon 2. The amplified fragment corresponding to native *PR3b* cDNA is 111bp in length (nt. 667–777 of the 888bp coding sequence), while that of the alternatively spliced transcript is 46bp in length (nt. 667–712 of the 771bp coding sequence; Fig. 3B). Therefore, a fragment of 65bp in length was deleted through the alternative splicing. To confirm whether the smaller fragment was a real spliced transcript of *PR3b* or an occasionally non-specific amplification product, we designed primers to amplify a longer fragment overlapping the previously amplified region and analysed the sequence details. The results revealed a consensus in the sequence observed to be alternatively spliced (Supplementary Fig. S2). As 65 is a non-integral multiple of three, the alternative splicing altered the reading frame as well as the amino acid sequence of PR3b and introduced a premature stop codon in the coding region of *PR3b* (Fig. 3B). This implies a potential functional alteration of PR3b caused by the alternative splicing. Intriguingly, two single-nucleotide changes were also observed, i.e. one missing T at position 688 and one extra A behind position 691 of the native *PR3b* (Fig. 3B). A following RACE (rapid amplification of cDNA ends) PCR was applied to determine whether other splicing events are present in the coding sequence of *PR3b*, using primers specific to the spliced junction of the spliced *PR3b* variant. Yet, no other splicing was observed (Supplementary Fig. S3).

To determine whether this was a consequence of transcription shifts of unidentified genes with homologous sequence to the splicing region of *PR3b* in tobacco, we amplified the corresponding genomic fragment using the same primers used for RT-PCR amplification. Figure 3C shows results of the comparison of RT-PCR and genomic DNA PCR amplification products of the wild type and the *nic1* mutant of Burley 21. This revealed that the genomic DNA PCR products from all of the plants were the same size as the native *PR3b* fragment and that no products corresponding to the alternatively spliced *PR3b* fragment were observed (Fig. 3C). This indicated that the 46bp fragment was an alternatively spliced product of *PR3b*. Furthermore, we observed that the truncated *PR3b* fragment was also present in wild-type tobacco at trace amounts (Fig. 3C) and that the amplification products of the native *PR3b* fragment were observed in the RT-PCR amplification products of low-nicotine mutants.

### Enzyme-specific activity of the spliced PR3b variant

**Fig. 4.**
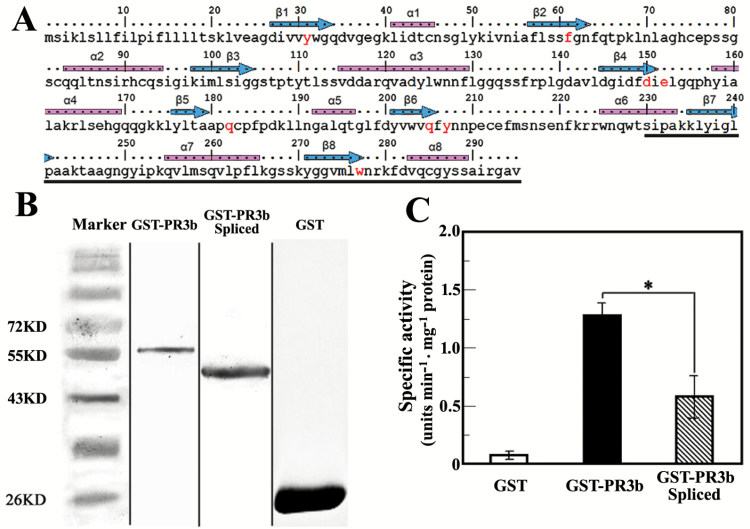
Specific chitinase activity of native and alternatively spliced PR3b. (A) Conserved α-helixes (blue arrows), β-sheets (pink bars), and amino acids (red characters) of PR3b compared with the catalytic cores of GH18 family chitinases (Hurtado-Guerrero and van Aalten, 2007). The region affected by alternative splicing is underlined with a black line. (B) Purified proteins of GST-tagged PR3b and GST separated by SDS-PAGE gel. ‘GST-PR3b’ is the GST-tagged protein of native PR3b; ‘GST-PR3b Spliced’ is the GST-tagged protein of spliced PR3b. Marker is the protein standard ruler. The picture shown is a combination of representative lanes from gels with serial elutions of the purified proteins. (C) Enzyme-specific activity of GST-tagged native and alternatively spliced PR3b. One unit equals 1 μmol of released 4-MU. GST is used for the control reaction. Values are the average of three replicates. Error bar, mean ±SD. The asterisk indicates a significant difference between the paired data sets (*P* <0.05, Student’s *t* test).

Tobacco PR3b is a class III plant chitinase, having a GH18 (glycosyl hydrolase family 18) domain with a pronounced active-site cleft at the C-terminal end of its beta-barrel (Hurtado-Guerrero and van Aalten, 2007; Tyler *et al.*, 2010). The alternative splicing of *PR3b* altered the amino acid sequence after Thr229 at its C-terminus which contains secondary structure regions (α6/7/8, β7/8) and an amino acid (Trp277) that are conserved across the GH18 chitinase family (Fig. 4A; Hurtado-Guerrero and van Aalten, 2007; Tyler *et al.*, 2010). Thus, the observed structural alterations suggested that a change in PR3b activity might have occurred. To determine this hypothesis, we expressed the wild-type PR3b protein and its spliced variant as GST-tagged fusions in *E. coli* BL21 cells, and the purified proteins (Fig. 4B) were tested for their chinolytic activity in a specific bioassay with GST as the control. Results showed that the enzyme-specific activity of native PR3b is about 2-fold higher than that of the spliced PR3b variant (Fig. 4C), suggesting that the alternative splicing of *PR3b* results in a significant reduction in the enzyme-specific activity of PR3b.

### Genetic linkage between enhanced PR3b splicing and the NIC loci in Burley 21

**Fig. 5.**
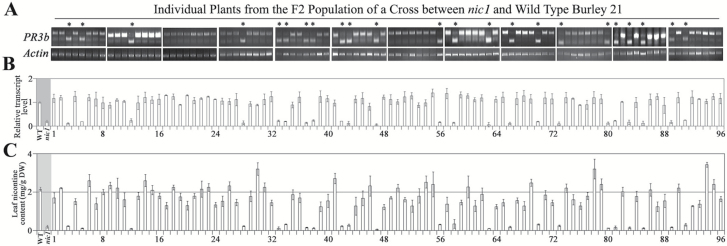
Genetic linkage between alternative splicing of *PR3b* and the *NIC1* locus in the Burley 21 background. (A) Alternative splicing of *PR3b* in individual F_2_ plants of a cross between *nic1* and wild-type Burley 21. (B) Transcript levels of *PMT1* in the roots of individual F_2_ plants. Transcript level of *PMT1* in the roots of wild-type Burley 21 was set as ’1’. *Actin* was used as an internal control. (C) Leaf nicotine content of individual F_2_ plants. The values shown are the means of three technical replicates. Error bar, mean ±SD.

Low-nicotine mutants *nic1* and *nic2* of Burley 21 were derived from the double mutant *nic1nic2* of Burley 21 (Legg and Collins, 1971), thus the coincident *PR3b* splicing in all these low-nicotine varieties somehow implies a linkage to the *NIC* loci. To explore the relationship between enhanced *PR3b* splicing and the *NIC* loci in Burley 21 further, we performed genetic analyses with an F_2_ population of the cross between the *nic1* mutant and wild-type Burley 21. In ~25% of the F_2_ plants (23/96), *PR3b* is alternatively spliced as it is in the *nic* mutants of Burley 21 (Fig. 5A), and the transcript levels of *PMT1* in these lines were also considerably lowered (Fig. 5B). Furthermore, the leaf nicotine content of the corresponding lines is very low (less than 0.35mg g^–1^ dry weight) compared with the other lines (Fig. 5C). We also analysed *PR3b* splicing in a small group of F_2_ plants of the cross between the *nic2* mutant and wild-type Burley 21 and obtained a similar result (Supplementary Fig. S4). These findings support a positive link between enhanced *PR3b* splicing and the *NIC* loci in Burley 21. On the other hand, the *NIC1* and *NIC2* loci are two unlinked loci (Legg *et al.*, 1969; Legg and Collins, 1971). The correlation between *PR3b* splicing and the *NIC* loci in Burley 21 implies the involvement of both *NIC1* and *NIC2* loci in regulating *PR3b* splicing.

### Regulation of PR3b splicing in the low-nicotine mutants of Burley 21 by phytohormones JA and ET

**Fig. 6.**
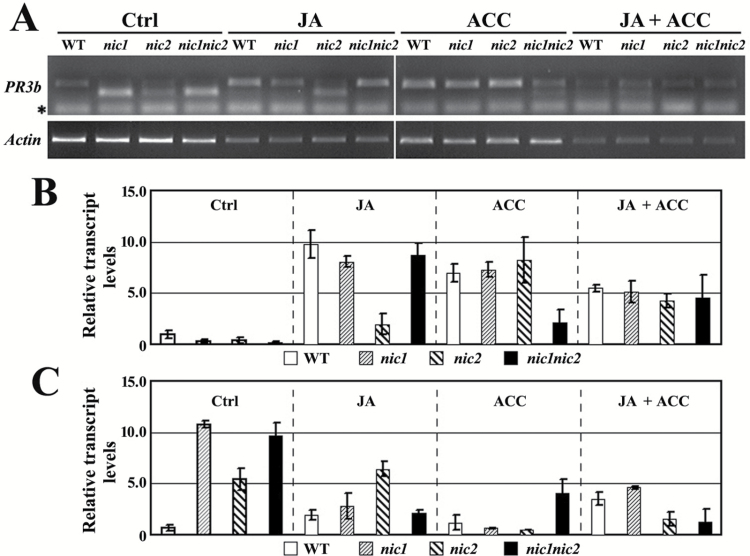
Phytohormone-induced splicing patterns of *PR3b.* (A) Splicing patterns of *PR3b* in the wild type (WT) and in low-nicotine mutants (*nic1*, *nic2*, and *nic1nic2*). The representative results of three independent replicates are shown. The asterisk indicates the primer dimers in the PCR amplification products. (B) Quantification of specific transcript of native *PR3b*. (C) Quantification of specific transcript of spliced *PR3b*. Ctrl indicates the untreated control; JA, ACC, and JA+ACC (the combination of JA and ACC) indicate different phytohormone treatments. The *PR3b* transcript level in the WT of the Ctrl treatment is set as ‘1’ (B, C). *Actin* was used as an internal control.

*NIC1* and *NIC2* are unlinked loci and they may display differential roles in regulating *PR3b* splicing. We noticed the induction of *PR3b* expression by JA and the precursor of ET which might be able to alter the regulatory effects of *NIC* loci on *PR3b* splicing. We then investigated the potentials of these two hormones on the alternative splicing of *PR3b* transcripts. The results showed that the alternative splicing patterns of *PR3b* were altered by treatments with JA, ACC, or the combination of JA and ACC (Fig. 6). JA treatment suppressed the alternative splicing of *PR3b* in *nic1* and *nic1nic2* mutants but not in *nic2*. By contrast, treatment with ACC or the combination of JA and ACC suppressed splicing in all *nic* mutants to different extents. Interestingly, the alternatively spliced fragment of *PR3b* was faintly visible in the amplification products of wild-type tobacco treated with JA or the combination of JA and ACC (Fig. 6A). The abundance of native and alternatively spliced *PR3b* was quantified by qRT-PCR amplification using specific primers. Our results showed that JA and/or ACC treatment could accentuate the transcript levels of native *PR3b* and attenuate the transcript levels of spliced *PR3b* in the low-nicotine mutants (Fig. 6B, C). These findings were similar to those observed in semi-quantitative RT-PCR. Taken together, our results suggest that the alternative splicing of *PR3b* is differentially regulated by JA and ET in the wild type and low-nicotine mutants of Burley 21 tobacco.

We also cloned the RT-PCR amplification products from each plant into the pBluescript II SK+ vector and sequenced them to determine the presence of the native and alternatively spliced transcripts. The sequencing analyses revealed that the major amplification products in the wild-type plants were the 111bp native fragments and that there were quite a few transcripts of the 46bp spliced fragment (Table 1). The major amplification products in the low-nicotine mutants were the 46bp spliced fragment, however, there were also considerable transcripts of the 111bp native fragment and their abundances were affected by phytohormone treatments (Table 1). These results are consistent with the results of the qRT-PCR assays and showed the presence of the spliced fragment in wild-type plants. This evidence indicates that mutations of the *NIC* loci altered the abundance of native and alternatively spliced *PR3b* transcripts in the low-nicotine mutants, i.e. mutation in *nic1* or *nic2* specifically enhanced the abundance of alternatively spliced *PR3b* transcripts.

**Table 1.** Abundance of native and alternatively spliced transcripts of *PR3b* in the RT-PCR amplification products of the roots of the wild type (WT) and in low-nicotine mutants of Burley 21

Treatment	Ctrl				JA				ACC				JA+ACC			
Genotype	WT	*nic1*	*nic2*	*nic1nic2*	WT	*nic1*	*nic2*	*nic1nic2*	WT	*nic1*	*nic2*	*nic1nic2*	WT	*nic1*	*nic2*	*nic1nic2*
**Native**	49	12	22	10	46	44	17	48	50	50	50	37	41	36	49	50
**Spliced**	1	38	28	40	4	6	33	2	0	0	0	13	9	14	1	0

Data were collected from a representative RT-PCR amplification of three independent replicates. Ctrl indicates the untreated controls. JA, ACC, and JA+ACC indicate the different phytohormone treatments. Native indicates the number of colonies containing native PR3b fragments. Spliced indicates the number of colonies containing alternatively spliced PR3b fragments.

## Discussion

By investigating the expression patterns of a set of PR protein genes in the low-nicotine mutants (*nic1*, *nic2*, and *nic1nic2*) of Burley 21 tobacco, we identified the basic chitinase gene *PR3b* as being alternatively spliced and then characterized this phenomenon in this study.

Tobacco PR3b belongs to the class III chitinase which has a conserved GH18 domain with eight-stranded β/α-barrel (Hurtado-Guerrero and van Aalten, 2007). The alternative splicing of *PR3b* mRNA caused a reading frame change and introduced a premature stop codon. Therefore, it changed the C-terminal amino acid sequence of PR3b and resulted in the loss of conserved domains and amino acid. And, this splicing could result in a reduction of the enzyme-specific activity of PR3b by half. PR3b has a functional role in tobacco resistance against fungal pathogens (Lawton *et al.*, 1992; van Loon *et al.*, 2006). Presumably, the enhanced alternative splicing of *PR3b* in the low-nicotine mutants might cause an alteration in the susceptibility to fungal pathogens. However, the transcription patterns of other PR protein genes were also altered in the *nic* mutants (Supplementary Fig. S5), which resulted in a certain difficulty in determining the specific change in anti-fungal capability caused by *PR3b* splicing. Two single-nucleotide changes (a missing T and an extra A) were also observed in the alternative spliced sequence of *PR3b*. Presumably, this was caused by nucleotide-deletion/insertion during mRNA splicing (Gott and Emeson, 2000). Yet, the cause of reading frame shift is the deletion of the 65bp fragment but not the single-nucleotide change.

In general pre-mRNA splicing, introns mostly start from GU, end with AG, and contain a so-called ‘branch site’ with the sequence CU(A/G)A(C/U) at about 20–50 bases upstream of the AG-end (Meyer *et al.*, 2015). Apparently, the *PR3b* splicing found in this study does not meet this rule. Alternative splicing is a complicated regulatory mechanism (Meyer *et al.*, 2015), and has been reported for several genes functioning in the plant response to pathogen attack (Dinesh-Kumar and Baker, 2000; Zhang and Gassmann, 2007). A previous finding that shares some similarities with this study is that of the alternative splicing of acidic chitinase II in *Citrus clementina*. This introduced a premature stop codon but the chitinase activity could be induced following MeJA treatment (Del Carratore *et al.*, 2011). Similarly, the enhanced alternative splicing of *PR3b* in the low-nicotine mutants could be suppressed by JA and/or ACC (the precursor of ET) treatments. Although the acidic chitinase II of *C. clementina* does not share any similarity with the basic chitinase PR3b in *N. tabacum*, they both provide evidence for JA-induced alternative splicing of chitinase, implying a common regulatory mechanism. These findings also provided evidence of JA/ET-signalling pathways in co-ordinating the alternative splicing of PR protein genes.

The low-nicotine mutants *nic1* and *nic2* were derived from LA Burley 21, i.e. the low-nicotine mutant *nic1nic2* of Burley 21 (Legg *et al.*, 1970; Legg and Collins, 1971). Hence, the coincident *PR3b* splicing patterns in all these low-nicotine varieties, to some extent, imply a linkage to the *NIC* loci. Consistently, the genetic analyses of F_2_ populations of crosses between wild-type Burley 21 and the *nic1* mutant suggested a positive link between *PR3b* splicing and the *NIC* loci in Burley 21. On the other hand, findings in this study showed that *PR3b* was spliced in both the wild type and the low-nicotine mutants of Burley 21, except that the spliced *PR3b* transcripts were enhanced to higher levels in the low-nicotine mutants of Burley 21. Thus, observation of enhanced *PR3b* splicing in these low-nicotine varieties suggests that *PR3b* splicing is co-ordinately regulated by the *NIC1* and *NIC2* loci, even though they are two unlinked loci (Legg *et al.*, 1970; Legg and Collins, 1971). Furthermore, the alternative splicing of *PR3b* in the low-nicotine mutants is differentially regulated by the phytohormones JA and ET. The alternative splicing of *PR3b* could be suppressed by ACC in all of the low-nicotine mutants to different extents but was only repressed by JA in *nic1* and *nic1nic2*. The difference in phytohormone-induced splicing patterns of *PR3b* in the low-nicotine mutants suggests that the *NIC1* and *NIC2* loci display differential roles in regulating *PR3b* splicing.

The regulation of alternative RNA splicing is an important part of the gene regulation network (Black, 2003; Reddy, 2007; Syed *et al.*, 2012; Yang *et al.*, 2014) which involves the regulation of stress and phytohormone responses (Palusa *et al.*, 2007; Reddy and Shad Ali, 2011). The finding of *PR3b* splicing regulation by JA/ET and *NIC* loci in Burley 21 is valuable to the genetic studies of low-nicotine mutants and could provide clues to unravel the mechanism by which JA/ET-signalling pathways regulate PR protein gene splicing.

## Supplementary data

Supplementary data can be found at *JXB* online.

Figure S1. Alignment of *PR3b* genomic sequence and CDS (coding sequence) amplified from tobacco Burley 21.

Figure S2. Sequence analysis of *PR3b* splicing region amplified with different primer sets.

Figure S3. Rapid amplification of cDNA ends (RACE) of alternatively spliced *PR3b*.

Figure S4. Alternative splicing of *PR3b* in the F_2_ population of a cross between *nic2* and wild-type Burley 21.

Figure S5. Phytohormone-induced transcription patterns of PR protein genes.

Supplementary Data
