# Supplementary material for: Alternative splicing of basic chitinase gene PR3b in the low-nicotine mutants of Nicotiana tabacum L. cv. Burley 21
Source: J Exp Bot. 2016 Sep 23;67(19):5799–809. doi: 10.1093/jxb/erw345 (PMC5066497; doi:10.1093/jxb/erw345)
Supplement: Supplementary Data [file supp_67_19_5799__index.html]

Alternative splicing of basic chitinase gene PR3b in the low-nicotine mutants of Nicotiana tabacum L. cv. Burley 21 — Alternative splicing of basic chitinase gene PR3b in the low-nicotine mutants of Nicotiana tabacum L. cv. Burley 21 — Supplementary Data 

# Alternative splicing of basic chitinase gene *PR3b* in the low-nicotine mutants of *Nicotiana tabacum* L. cv. Burley 21

## Supplementary Data

Data files

- supplementary\_figures\_S1\_S5.pdf - Supplementary Data
