# Supplementary material for: Alternative splicing of basic chitinase gene PR3b in the low-nicotine mutants of Nicotiana tabacum L. cv. Burley 21
Source: J Exp Bot. 2016 Sep 23;67(19):5799–809. doi: 10.1093/jxb/erw345 (PMC5066497; doi:10.1093/jxb/erw345)
Supplement: Supplementary Data [file supp_erw345_supplementary_figures_S1_S5.pdf]

Supplementary Figures

```
Genomic seqence    1 atgagcattaagctatctttacttttcattctaccaatattcttgcttctcctaaccagcaaattagtagaagctggagatactgtggtctattggggccaagatgtaggagaaggtaaa
    CDS seqence    1 atgagcattaagctatctttacttttcattctaccaatattcttgcttctcctaaccagcaaattagtagaagctggagatactgtggtctattggggccaagatgtaggagaaggtaaa

Genomic seqence  121 ttgattgacacatgcaactctggtctctacaaaattgtcaacattgcctttttatcttcttttggcaatttccaaactcctaaacttaacttagctggccattgtgaaccatcttctggt
    CDS seqence  121 ttgattgacacatgcaactctggtctctacaaaattgtcaacattgcctttttatcttcttttggcaatttccaaactcctaaacttaacttagctggccattgtgaaccatcttctggt

Genomic seqence  241 agttgccaacagttgacaaacagcattagacattgtcaaagcataggaattaaaatcatgctctccattggaggttcaactcctacctacacattatcctcagttgatgatgctaggcaa
    CDS seqence  241 agttgccaacagttgacaaacagcattagacattgtcaaagcataggaattaaaatcatgctctccattggaggttcaactcctacctacacattatcctcagttgatgatgctaggcaa

Genomic seqence  361 gttgctgattacctatggaacaattttctcggcggccaatcatcttttaggccacttggagatgctgtgctagatggcatagattttgacattgaacttggccaaccgcattatattgca
    CDS seqence  361 gttgctgattacctatggaacaattttctcggcggccaatcatcttttaggccacttggagatgctgtgctagatggcatagattttgacattgaacttggccaaccgcattatattgca

Genomic seqence  481 cttgccaagaggctttcagaacatggccaacaaggtatgtcaaacagactttttattaaagccgaacagaggtggaaccaggatttgaaacatatatgttcaatattctgattctttta
    CDS seqence  481 cttgccaagaggctttcagaacatggccaacaa---------------------------------------------------------------------------------------

Genomic seqence  601 agttaataatttgtgcacaattcaataacttttttaagacaaatacagagtttggacaaaagttactgggttcagccgaacccatacccaaagggctagctccgcccctgaagccaaagga
    CDS seqence      ------------------------------------------------------------------------------------------------------------------------

Genomic seqence  721 tggatatcatgatgtgttgtagaataaccaagaattgcagtatcccatgttaaccaagcacaaagaaaataattgtaatttggttgttgaataggttgaaggggagcctttgcgtaactg
    CDS seqence      ------------------------------------------------------------------------------------------------------------------------

Genomic seqence  841 gtaaagttattttcacgtgactaggaggtcatgaattcgagccttggaaatagcctcttgtaaaaatgcagggtgagactgtatacaatagatccttgtggttcgacccttctactccgc
    CDS seqence      ------------------------------------------------------------------------------------------------------------------------

Genomic seqence  961 gcattagcgggagcttaatgcaccgagttgtctttttttgttgttgttgaatgcaacactttggttcagtggtaagtacagaacacctgatgcattggttaattagacgaacatcactgg
    CDS seqence      ------------------------------------------------------------------------------------------------------------------------

Genomic seqence 1081 tttgagccctgccacagagatatttggtaattaagttggaagaagggtaaaagggcgagttcaaacactgtgagccaactggtgtcgggccagaagcgcgcagggaatttctccggaaga
    CDS seqence      ------------------------------------------------------------------------------------------------------------------------

Genomic seqence 1201 aatgttctctgatataaacttgtgcactaactaatatcgtgccagaagcccacgagggatttctccaacataaaatgaaaatgtcattaaacatcaattctagattttcaagccattaga
    CDS seqence      ------------------------------------------------------------------------------------------------------------------------

Genomic seqence 1321 gtggcaaaactcattctcttattatgtcttttgaccggtaaaaaaatatacttaactgcagcaccacaatgtccttttcctgataaacttcttaacggtgcattacaaactggcttattt
    CDS seqence  514 ------------------------------------ggtaaaaaaatatacttaactgcagcaccacaatgtccttttcctgataaacttcttaacggtgcattacaaactggcttattt

Genomic seqence 1441 gactatgtttgggtccaattttacaacaatcccgagtgcgagtttatgagcaattcagaaaatttcaagaggaggtggaatcagtggacatcaatccctgcaaagaagttgtatattgga
    CDS seqence  598 gactatgtttgggtccaattttacaacaatcccgagtgcgagtttatgagcaattcagaaaatttcaagaggaggtggaatcagtggacatcaatccctgcaaagaagttgtatattgga

Genomic seqence 1561 cttccagcagccaaggcagccgcgggtagtggctatattccaaagcaagtgctaatgtcacaagttttaccatttctaaagggatcttcaaagtatggaggtgtcatgctttggaataga
    CDS seqence  718 cttccagcagccaaggcagccgcgggtagtggctatattccaaagcaagtgctaatgtcacaagttttaccatttctaaagggatcttcaaagtatggaggtgtcatgctttggaataga

Genomic seqence 1681 aaatttgatgttcaaagtggctatagctctgctatcaggggtgctgtttaa
    CDS seqence  838 aaatttgatgttcaaagtggctatagctctgctatcaggggtgctgtttaa
```

Fig. S1. Alignment of *PR3b* genomic sequence and CDS (coding sequence) amplified from tobacco Burley 21.

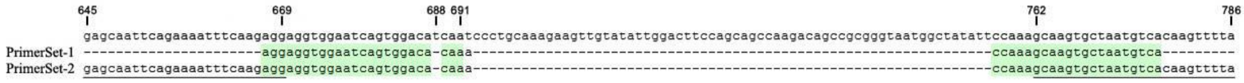

Fig. S2. Sequence analysis of *PR3b* splicing region amplified with different primer sets.

Top line shows the sequence of native *PR3b*. Numbers over native *PR3b* sequence indicates nucleotide positions in the full-length coding sequence. PrimerSet-1 indicates the sequence amplified with primers mentioned in the main text for detection of both native and spliced *PR3b*. The sequence labeled as PrimerSet-2 was amplified with primers according to the underlined sequences.

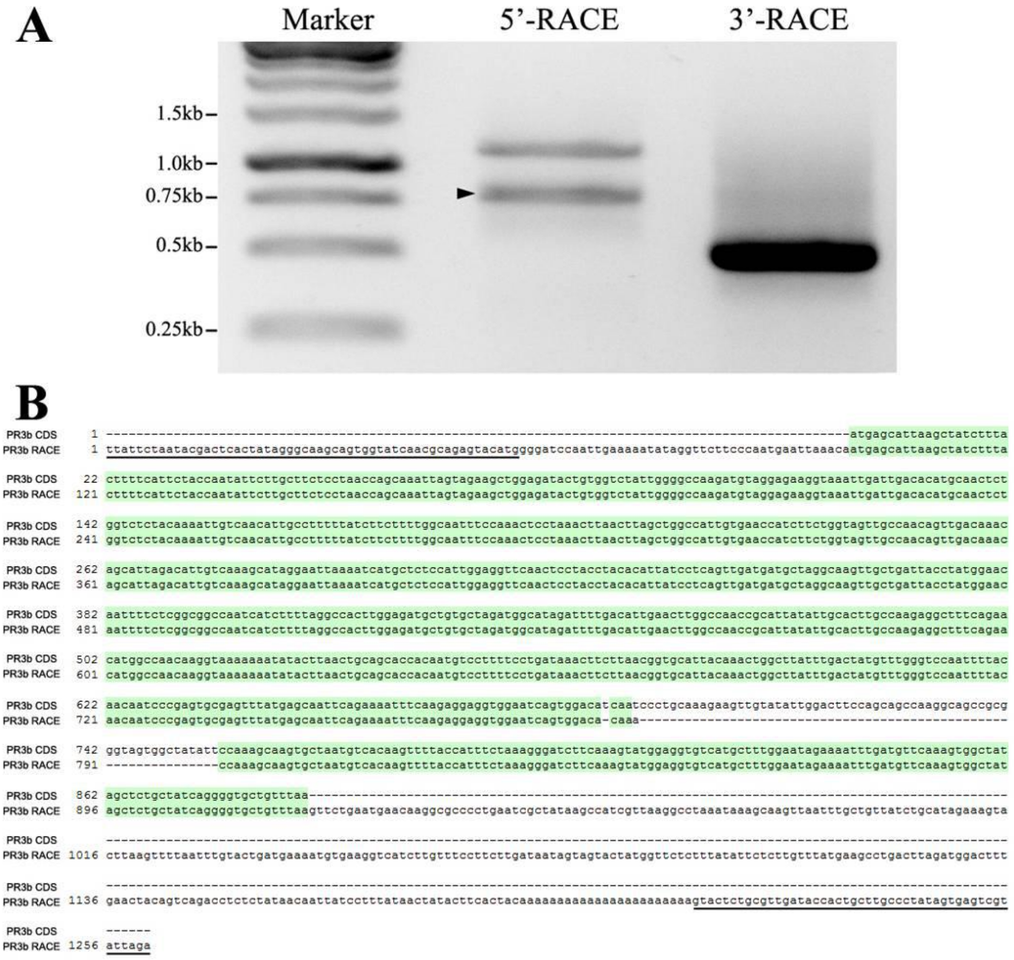

Fig. S3. Rapid amplification of cDNA ends (RACE) of alternatively spliced *PR3b*. (A) Electrophoresis of RACE PCR products. Triangle in the lane of 5'-RACE indicates the band containing target RACE PCR product. (B) Sequence alignment of spliced *PR3b* obtained by RACE PCR and the coding sequence of native *PR3b*. Underlined sequences indicate amplified adapters in the universal primers of SMARTer RACE kit.

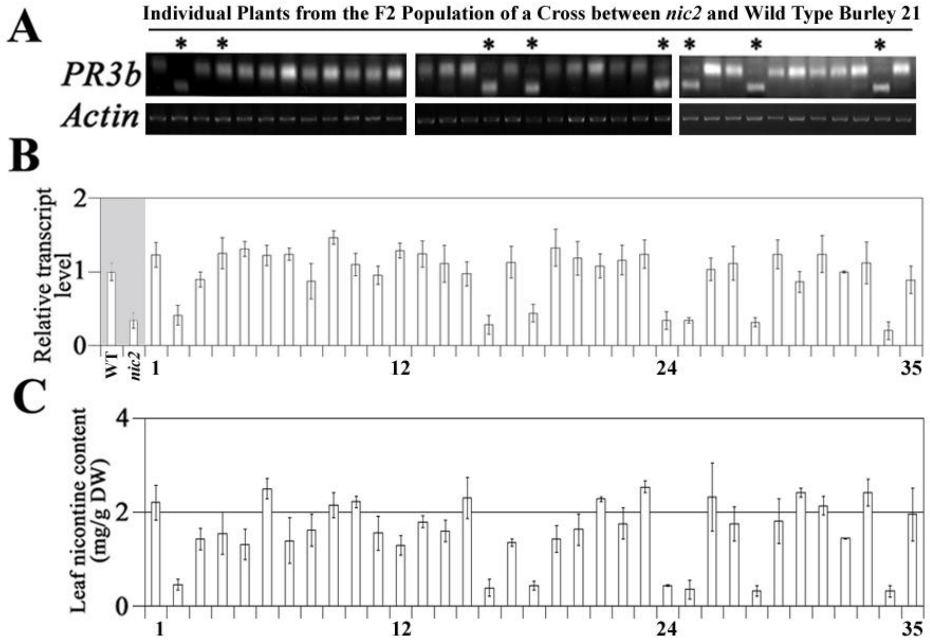

Fig. S4. Alternative splicing of *PR3b* in the F2 individuals of a cross between *nic2* and wild type Burley 21. (A) Alternative splicing of *PR3b* in individual F2 plants. (B) Transcript levels of *PMT1* in the roots of individual F2 plants. Transcript level of *PMT1* in the roots of wild type Burley 21 was set as “1”. *Actin* was used as an internal control. (C) Leaf nicotine content of individual F2 plants. Shown values are means of three technical replicates. Error bar, mean ± SD.

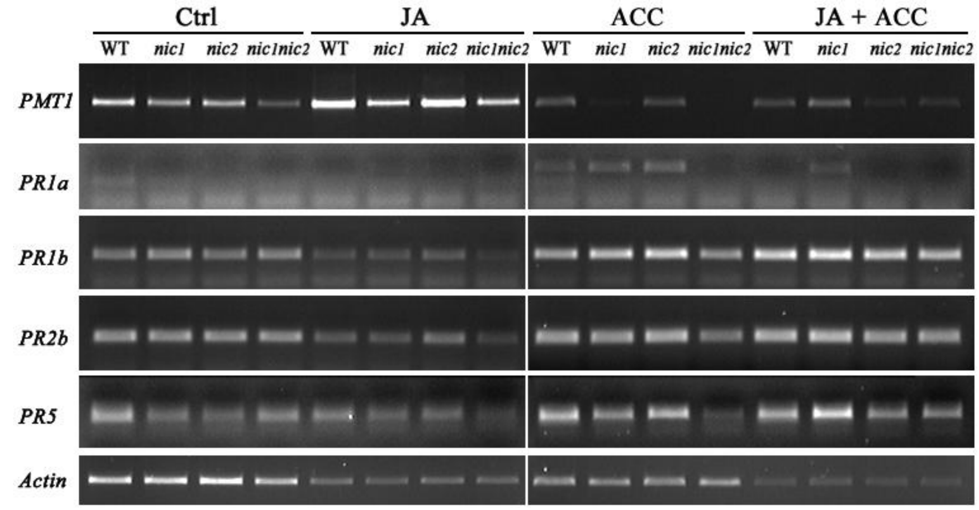

Fig. S5. Phytohormone-induced transcription patterns of PR protein genes. The transcription patterns of PR protein genes in wild type (WT) and low-nicotine mutants (*nic1*, *nic2*, and *nic1nic2*) were analyzed following treatment with JA, ACC, or the combination of JA and ACC (JA+ACC). Shown are representative results of three independent replicates. Ctrl indicates the untreated controls. *Actin* was used as an internal control.
